# The Evolution of Kidney Graft Preservation Through the Years

**DOI:** 10.3390/life14121647

**Published:** 2024-12-11

**Authors:** Andres Calva Lopez, Jose Enrique Robles Garcia, Carlos Andres Yanez Ruiz, Mario Daniel Tapia Tapia, Vanessa Talavera Cobo, Carmina Alejandra Muñoz Bastidas, Daniel Sanchez Zalabardo, Bernardino Miñana Lopez

**Affiliations:** Department of Urology, Clínica Universidad de Navarra, 31008 Pamplona, Spain

**Keywords:** kidney transplantation, kidney graft preservation, static cold storage, hypothermic machine perfusion, delayed graft function

## Abstract

Chronic kidney disease (CKD) is a prevalent disease affecting almost 10% of the world’s population, with many cases progressing to end-stage kidney disease (ESKD). Kidney transplantation (KT) is the gold-standard treatment for ESKD. Due to growing KT waitlists, the deceased kidney donor (DKDs) criteria have expanded to increase the number of available kidney grafts. Kidney graft preservation ensures optimal graft function after KT. Static cold storage (SCS) as a preservation method is still widely used. Hypothermic machine perfusion (HMP) has proven to decrease delayed graft function (DGF) and increase graft survival. Most recent studies advocate for the use of HMP regardless of donor type. However, emerging technologies, such as hypothermic oxygenated machine perfusion (HOPE) and normothermic machine perfusion (NMP), have shown promising results in specific scenarios. This review aims to provide a summary of the well-established kidney graft preservation methods and their outcomes, as well as novel technological advances that allow for newer preservation strategies.

## 1. Introduction

Chronic kidney disease (CKD) affects more than 800 million people worldwide, about 10% of the world’s population [1]. If left untreated, it eventually evolves to end-stage kidney disease (ESKD), requiring renal replacement therapy (RRT). In 2015, a series of Global Burden of Disease (GBD) studies placed CKD as one of the leading causes of mortality worldwide, clearly stating the urgent need for not only early diagnosis and treatment, but also the establishment of preventive measures [2]. When it evolves to ESKD, RRT takes a big toll on national health systems and patient morbimortality. As of today, the gold-standard RRT for ESKD is kidney transplantation (KT), as it improves overall patient survival, reduces the morbidity associated with other forms of RRT (e.g., hemodialysis), improves patient quality of life and is a cost-effective measure for health systems worldwide. In 2021, in the United States alone, more than 25,500 KTs were performed [3]. In Europe, the ERA Registry published data in 2023, with record-breaking numbers of KT, from 29.6 KT per million population (pmp) in 2010 to 34.7 KT pmp in 2018, with an estimated annual increase of almost 2% [4]. This suggests that the number of KTs will continue to increase in the upcoming years; however, waitlisted ESKD patients continue to increase at an exponential rate. The greatest obstacle to reducing waitlist numbers is the limited number of deceased kidney donors (DKDs), and as the worldwide population ages, the quality of these grafts becomes suboptimal. It is of the highest importance to guarantee kidney grafts are adequately preserved during and after organ procurement, to safeguard renal function and ensure the best possible results for KT recipients.

Until recent years, the standard DKD pool was composed of donors after brain death (DBDs), defined as donors that meet neurological brain-stem death criteria. The continuing shortage of DKDs and increasing waitlists of ESKD patients led to the need to consider potential donors that did not meet the DBD criteria, with a new group of DKDs being implemented, known as donors after circulatory death (DCDs), allowing for an exponential increase in the number of kidney grafts donations worldwide. Nonetheless, DKD numbers still lagging behind ESKD waitlisted patients, and a continuously aging population permitted the introduction of expanded-criteria donors (ECDs), defined as donors > 60 years, or between 50 and 59 years, with at least 2 of the following criteria: a history of hypertension, death resulting from stroke, or serum creatinine levels ≥ 1.5 mg/dL prior to donation.

Kidney graft preservation techniques have customarily relied on hypothermia, allowing a reduction in kidney metabolism and the hinderance of enzymatic tissue degradation by almost 3-fold for every 10 °C reduction in graft temperature. This limits the degree of adenosine triphosphate (ATP) depletion and inhibits its degradation, allowing for more prolonged tissue survival [5]. Nevertheless, regardless of these mechanisms, cold ischemia degrades viable renal tissue due to a gradual depletion of basic substrates for adequate cell survival. This occurs through a series of metabolic changes: intracellular acidosis through the production of lactic acid as a byproduct of anaerobic metabolism; decreased Na^+^/K^+^ ATPase pump activity, allowing a disbalance in Na^+^ and K^+^ ions which produce intracellular edema; damage and dysregulation of the cell cycle, causing cellular apoptosis and necrosis [6,7]. These molecular cascades are augmented during reperfusion, further damaging viable renal tissue, in what is known as ischemia–reperfusion injury, with the triggering of inflammatory and oxidative pathways [7]. Multiple studies have identified cold ischemia time (CIT) in itself as a prognostic independent factor for the development of delayed graft function (DGF), defined as the need for hemodialysis during the first 7 days after transplantation. The risk of DGF increases by 10% for every hour of CIT [8], irrespective of the type of donor, as well as enhancing graft immunogenicity, which results in acute kidney injury (AKI) and the induction of chronic allograft rejection [9,10]. Grafts from all donor types are susceptible to cold ischemia and its consequences, which justifies implementing different preservation methods in an attempt to reverse or minimize the adverse effects of cold ischemia.

In this review, we will attempt to provide a comprehensive assessment of the achievements in kidney graft preservation, different preservation methods, and evolving techniques that have allowed successful transplantation programs to endure the increasing demand of rising KT numbers while improving functional results.

## 2. Static Cold Storage

**Figure 1:**
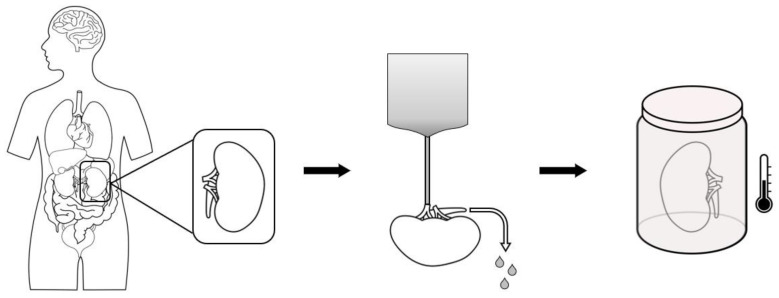
Steps for static cold storage (SCS) organ preservation.

Static cold storage (SCS) is a simple, widely spread and well-known kidney preservation method. It consists of a series of phases: flushing residual blood of the kidney graft, rapidly reducing graft tissue temperature, and storage within a sterile container while submerged in a specific preservation solution (PS) surrounded by ice to maintain a stable temperature from 0 °C to 4 °C (Figure 1). SCS is an inexpensive, easy-to-use and readily available preservation method that has allowed for kidney graft preservation worldwide.

PS composition is what allows SCS to preserve graft function and viability, regardless of the damage produced by cold ischemia. Generally speaking, PS entails three key components that allow for adequate preservation: an impermeant that responds to edema and provides cellular structure stability; a buffer solution to counteract the accumulation of intracellular acidosis; a balanced electrolyte composition (either a low or high Na^+^/K^+^ ratio) to prevent edema [11].

For DKD KT, SCS is the most popular preservation method, due to its widespread use, simplicity and low cost. The most commonly used PSs for DKD KT include University of Wisconsin (UW) solution (Bridge to Life Ltd., Columbia, SC, USA), developed by Belzer in the 1980s, containing antioxidants to neutralize oxygen free radicals, adenosine (an ATP precursor) [12] and a colloid to counteract tissular edema, and Celsior solution (Institut Georges Lopez, Lissieu, France), originally designed for heart preservation and transplantation but now widely used for kidney graft preservation, containing a buffer to prevent acidosis and mannitol to counteract edema and antioxidants [13]. There is conflicting evidence on which PS works best, with most studies arguing that PSs are equivalent regarding DGF rates [14,15]. Only a single prospective study found higher 2-year graft survival using Celsior when compared to UW [16]. For living donor (LD) KT, SCS is the preferred preservation method due to the very short warm ischemia times (usually below 10 min) and generally reduced CIT. Experienced LD transplantation centers can often neglect graft preservation by SCS and proceed to implantation immediately after procurement.

## 3. Hypothermic Machine Perfusion

**Figure 2:**
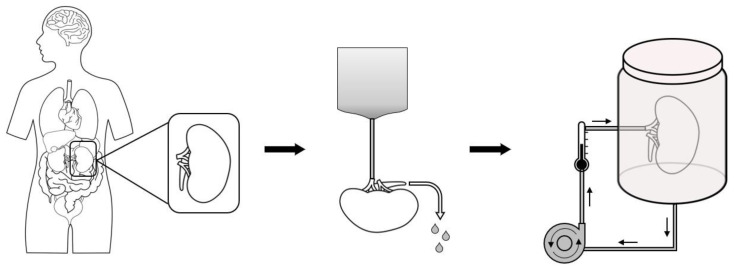
Steps for hypothermic machine perfusion (HMP) organ preservation.

Hypothermic machine perfusion (HMP) is a not-so-recent preservation method that consists of a continuous or pulsatile circuit of perfusion while maintaining a low temperature (2 °C to 8 °C), allowing the complete washout of residual blood and metabolites, and consequent graft stasis with the perfusion solution (Figure 2). Just like SCS, the perfusion solution consists of different components that protect the graft and delivers basic substrates for normal renal metabolism to endure while under cold ischemia.

**Figure 3:**
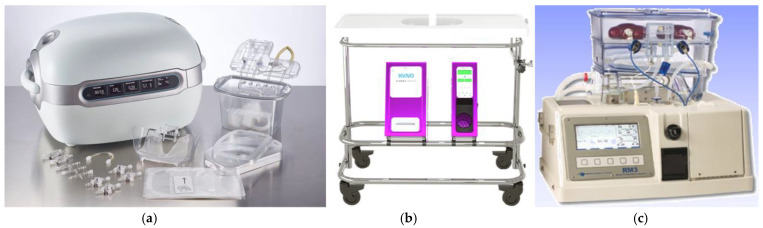
(**a**) LifePort^®^ Kidney Transporter; (**b**) Kidney Assist^TM^; (**c**) RM3^®^.

HMP dates back to the 1960s, enabling the preservation of canine kidneys for up to 72 h by Belzer et al. in 1967 [17], which laid the groundwork for the same group to successfully perform the world’s first HMP-preserved DKD KT a year later [18]. The last 30 years have witnessed fast-tracked technological advances in HMP, resulting in a variety of commercially available HMP systems. Nowadays, some of the most widespread and commonly used HMP systems include the LifePort^®^ Kidney (Organ Recovery Systems, Chicago, IL, USA) Transporter (Organ Recovery Systems), the Kidney Assist^TM^ (Organ Assist, Gothenburg, Sweden)and the RM3^®^ (Waters Medical Systems, Rochester, MN, USA) (Figure 3). These types of second-generation HMP systems allow for the assessment of real time parameters during graft preservation, including perfusion pressure (systolic and diastolic), flow, resistance and temperature. The main difference between these systems is that the LifePort^®^ Kidney Transporter and the Kidney Assist^TM^ systems are pressure-driven HMP systems, while the RM3^®^ is a flow-driven HMP system. A unique characteristic of the Kidney Assist^TM^ HMP system is its capability to also provide normothermic machine perfusion (NMP).

### 3.1. How Does HMP Work?

The pulsatile perfusate flow through a kidney graft, which mimics physiological vascular perfusion, protects the endothelial cellular membrane from depolarization and reduces the formation toxic free radicals [11], while increasing the endogenous production of nitric oxide and Krüpper-like factor 2, an endothelial anti-inflammatory transcription factor, and also reducing levels of endothelin-1 [19]. This allows for generalized microvasculature vasodilation, reducing perfusion resistance parameters and ultimately improving graft viability. At the same time, molecular studies have determined that HMP reduces levels of proinflammatory cytokines and induces Akt-Erk pathway phosphorylation, which has been linked to less tissular inflammation, less oxidative damage, electrolyte homeostasis, and ultimately, a decrease in cellular apoptosis [20,21].

HMP parameter configuration allows for perfusion modification throughout the preservation process. Perfusion pressure can be increased or decreased to ensure proper perfusion and preservation. Evidence suggests that low-pressure HMP settings benefit graft preservation and function. Animal autotransplantation models have proven that high-pressure HMP settings (60/40 mmHg) compared to low-pressure HMP settings (30/20 mmHg) yield higher levels of proinflammatory cytokines and von Willebrand factor, adding to the already existing endothelial damage due to cold ischemia, leading to an increased risk of graft thrombosis [22]. HMP systems can be pressure-driven (PD) or flow-driven (FD). A prospective, randomized controlled trial (RCT) evaluated 1-year results by comparing a PD-HMP system (LifePort^®^ Kidney Transporter) and an FD-HMP system (RM3^®^); although there were no differences in DGF, the 1-year graft survival rates enabled a PD-HMP preservation strategy. FD-HMP systems require almost a 50% higher systolic pressure to maintain an equal flow compared to PD-HMP systems, producing endothelial damage and augmenting the risk of graft thrombosis, which determines graft survival [23].

### 3.2. Effect of HMP

As evidence emerged, HMP displaced SCS as the preferred preservation method in many KT programs worldwide. Multiple studies have compared HMP to SCS, particularly with regard to the incidence of DGF. The Eurotransplant International Foundation published one of the largest RCTs comparing SCS with HMP with 672 kidney grafts from 336 DKDs (87.5% DKDs were DBDs and only 28% were ECDs), finding that HMP reduced the risk of DGF when compared to SCS by 43% (odds ratio = 0.57; *p* = 0.01). They found 1-year graft survival to be superior with HMP compared to SCS (94% vs. 90%; *p* = 0.04) [24]. The group performed a follow-up and published similar findings, with superior 3-year graft survival with HMP (91% vs. 87%; adjusted hazard ratio = 0.60; *p* = 0.04). Subgroup analysis still displayed similar 3-year survival results for DBDs (91% vs. 86%; adjusted hazard ratio = 0.54; *p* = 0.02) and ECDs (86% vs. 76%; adjusted hazard ratio = 0.38; *p* = 0.01), but surprisingly not in DCDs. They also concluded that regardless of the preservation method, HMP or SCS, the presence of DGF had a profound effect on graft survival in DBDs [25]. Treckmann et al. published the results of only ECDs from the main data set produced by the Eurotransplant trial [24], finding that HMP reduced the incidence of DGF when compared to SCS by more than 50% (adjusted odds ratio = 0.46; *p* = 0.047) and significantly higher 1-year death-censored graft survival with HMP (92% vs. 80%; *p* = 0.02). Similarly to the original data set, when only considering ECD grafts that developed DGF, 1-year graft survival significantly decreased when HMP was not performed (85% vs. 41%; *p* = 0.003) [26]. From the Scientific Registry of Transplant Recipients data, Gill et al. analyzed data from 94,709 KT recipients (SCDs, *n* = 71,192; ECDs, *n* = 15,122; DCDs, *n* = 8395) in the United States from 2000 and 2011, with similar results, associating HMP with a reduced incidence of DGF in all donor types (SCD odds ratio = 0.69; ECD odds ratio = 0.59; DCD odds ratio = 0.80). Even when considering CIT, the results remained consistent, with lower adjusted odds of DGF with HMP when compared to SCS, at each specific CIT strata [27]. While these results support the superiority of HMP over SCS, longer CIT entails an increased risk of DGF, irrespective of the preservation method used or the donor type.

A Cochrane meta-analysis, which included data from 2266 KT recipients from 16 RCTs, found that irrespective of donor type, HMP reduced the incidence of DGF when compared to SCS (relative risk = 0.77; *p* = 0.0006). When stratifying by donor type, HMP still reduced the incidence of DGF in DBDs (relative risk = 0.75; *p* = 0.0002) and DCDs (relative risk = 0.78; *p* = 0.006), in all cases with high certainty evidence [28]. Another systematic review and meta-analysis with data from 2048 KT recipients from 13 RCTs found similar results, with a reduced incidence of DGF irrespective of donor type (relative risk = 0.78; *p* < 0.0001), in DBDs (relative risk = 0.78; *p* = 0.003) and in DCDs (relative risk = 0.73; *p* = 0.001). This meta-analysis also concluded that HMP increased 1-year and 3-year graft survival [29]. In 2024, Tingle et al. updated their results from their first Cochrane systematic review and meta-analysis, with data from 4007 KT recipients from 22 RCTs, concluding that HMP reduces the rate of DGF when compared to SCS (relative risk = 0.78; *p* < 0.0001) [30].

## 4. Therapeutic Hypothermia

Therapeutic hypothermia (TH), also known as mild therapeutic hypothermia or targeted temperature management, consists of reducing a patient’s body temperature to a target of 32 °C to 35 °C. TH is a recognized procedure by international resuscitation guidelines, reducing mortality and neurological outcomes after out-of-hospital cardiac arrest. Although its effect on renal function protection remains uncertain, TH seems to have a protective effect [31]. Animal studies have found preserved renal function in rabbits after cardiac arrest compared to control groups, regardless of inflammatory or immune responses triggered by ventricular fibrillation or resuscitation [32]. An RCT of DBDs undergoing TH or normothermia before kidney graft procurement found a lower incidence of DGF with the use of TH (28% vs. 39%; *p* = 0.008). However, when comparing TH with HMP, its protective effect was not superior to that of HMP. Malinoski et al. found TH to be inferior to HMP in DBDs regarding DGF, irrespective of combining TH with HMP of procured kidney grafts (30% in TH group, 19% in HMP group, 22% in combined-therapy group), although 1-year graft survival remained similar between groups [33].

## 5. Novel Preservation Methods

SCS and HMP are well-established kidney graft preservation methods. HMP has substantially challenged established preservation logistics, providing more than satisfactory results when compared to SCS with regard to DGF and graft survival. Nevertheless, it comes with some disadvantages (Table 1). Technological advances have allowed for newer preservation methods to arise, with the aim of overcoming the difficulties posed by SCS and HMP.

### 5.1. Hypothermic Oxygenated Machine Perfusion

Hypothermic oxygenated machine perfusion (HOPE) involves supplemental oxygenation to standard HMP. Although mostly studied and applied for liver preservation, it has become an increasingly popular kidney graft preservation method. Oxygen can be bubbled into the perfusion solution to maintain stable partial pressure or added by an external membrane oxygenator, reaching higher oxygen partial pressures. Early studies advocated for short periods of oxygen supplementation during HMP, with improved graft function and reduced cytokine levels and immune responses [34,35]. The COMPARE study, a multicenter European RCT, compared HMP with HOPE in DCD grafts and found no differences regarding the 1-year estimated glomerular filtration rate (primary endpoint), but found that HOPE grafts developed fewer complications, less biopsy-proven acute rejection and lower rates of 1-year graft loss (secondary endpoints) [36]. Similar results have been published in recent years, finding no differences regarding DGF [37], with some arguing that longer HOPE (more than 2 h) does reduce the incidence of DGF compared to shorter HOPE (0% vs. 40%; *p* = 0.04) [38]. More RCTs are warranted to accurately establish the effect oxygen supplementation has on kidney graft preservation.

### 5.2. Normothermic Machine Perfusion

Normothermic machine perfusion (NMP) seeks to enable graft perfusion at normal temperatures. The reinstatement of normothermic conditions in kidney grafts, instead of metabolism suppression, promotes aerobic cellular metabolism and induces cellular repair pathways, restocking ATP levels, imitating an almost physiological environment for graft preservation [39]. A disadvantage of NMP is the upregulation of inflammatory pathways as a result of metabolism restoration. Preclinical evidence suggests that suboptimal or marginal kidney grafts, specially from ECDs, benefit from a short periods of NMP prior to transplantation (end-ischemic NMP), restoring graft function, which would otherwise be unnecessarily discarded [40]. However, various clinical studies have found conflicting evidence on the incidence of DGF when compared to SCS or HMP [41], with only one study advocating for the superiority of end-ischemic NMP compared to SCS [42]. Further research on end-ischemic and continuous NMP is needed to establish its impact on graft preservation its and possible clinical applications.

## 6. Conclusions

Kidney graft preservation has evolved in the last 50 years. Today, there are a number of preservation techniques, each with its advantages and disadvantages (Table 1). SCS still remains a valid strategy, especially in LD KT programs. The arrival of HMP revolutionized graft preservation and logistics for KT programs worldwide. Evidence clearly situates HMP as superior compared to SCS, in both DGF and graft survival, independent of donor type. Emerging technologies have allowed modifications to the well-established HMP, such as oxygen supplementation (HOPE) or normothermic preservation (NMP), showing promising initial results. However, its technical and logistical difficulties still prevent its widespread implementation in KT programs worldwide. The number and commercial availability of different kidney graft preservation techniques continues to grow, and they are likely to become tailored to specific donor types and clinical situations, thus increasing the number of available and viable kidney grafts worldwide.

## Figures and Tables

**Table 1:** Advantages and disadvantages of different types of kidney graft preservation strategies, and the most benefited type of donor for each preservation strategy.

Preservation Method	Advantages	Disadvantages	Preferred Type of Donor
SCS	Low cost; reduces metabolism; simple transportation	Depletion of ATP/substrates; time-limiting	LD, DBD
HMP	Reduces metabolism; simple transportation; assessment of graft quality	Technical and logistical difficulties	DCD, ECD
HOPE	Assessment of graft quality; oxygen/substrate supplementation	Technical and logistical difficulties; personnel training	DCD
NMP	Maintains metabolism; assessment of graft quality; oxygen/substrate supplementation; upregulation of repair pathways; reduces cold ischemia injury	Technical and logistical difficulties; high cost; upregulation of inflammatory pathways; personnel training	ECD

## Data Availability

Not applicable.
